# Context-Dependent Significance of London Dispersion

**DOI:** 10.1021/acs.accounts.3c00625

**Published:** 2023-11-23

**Authors:** Louis-Albin Gravillier, Scott L. Cockroft

**Affiliations:** EaStCHEM School of Chemistry, University of Edinburgh, Joseph Black Building, David Brewster Road, Edinburgh EH9 3FJ, U.K.

## Abstract

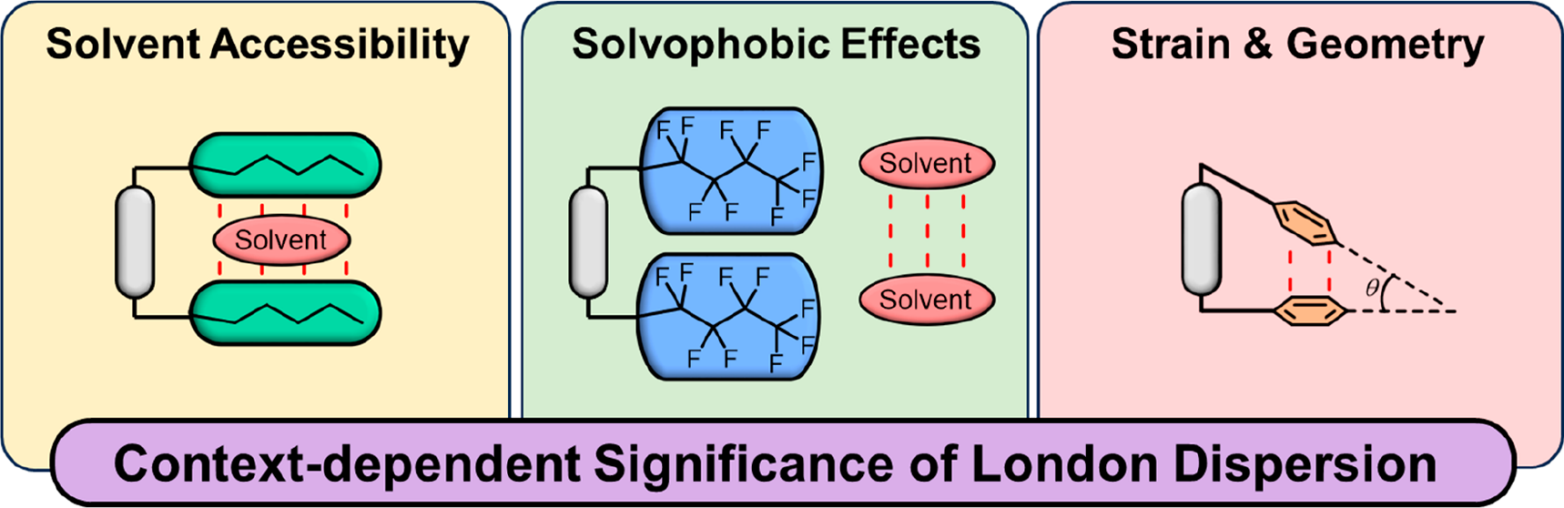

London forces constitute an attractive component of van der Waals interactions and originate from transient correlated momentary dipoles in adjacent atoms. The in-depth investigation of London dispersion forces poses notable challenges, especially in solution, owing to their inherently weak and competing character. Our objective in this Account is to shed light on the context-dependent significance of London dispersion forces by contrasting our own experimental findings with those from other research endeavors. Specifically, we will explore how factors such as the choice of system and solvent can influence the apparent role of London dispersion forces in molecular recognition processes. We initiate our Account by scrutinizing the Wilcox balance, which has yielded diverse and occasionally contradictory results. Following that, we provide an overview of the role of London dispersion forces and their context-dependent variations, encompassing alkyl–alkyl, halogen−π, alkyl−π, and aromatic stacking interactions.

Several experimental investigations have revealed how difficult it is to measure the significance of London dispersion in solution. Indeed, dispersion forces seldom act as the exclusive driving force in molecular recognition processes, and solvation energetics also strongly influence equilibria and kinetics. Molecular balances that bring apolar functional groups into contact have proven to be instrumental in the experimental measurement of dispersion. The intramolecular approach avoids the need to pay the entropic cost of bringing interacting groups into contact, while also enabling solvent screening. Such experimental studies have found dispersion interactions between functional groups to be very weak (<5 kJ mol^–1^), meaning that they frequently take backstage to electrostatic contributions and solvophobic effects and are readily damped by competitive dispersion interactions with the solvent. By using such approaches, competitive dispersion interactions with the solvent have been shown to be described by the bulk polarizability of the solvent (perfluoroalkanes have the lowest bulk polarizabilities, while carbon disulfide has one of the highest). Dispersion interactions are also strongly distance-dependent, which results in considerable context-dependent outcomes across different investigations. For example, we caution against the risk of attributing the stability of a “more sterically hindered” isomer as arising from intramolecular dispersion forces. The total energy of the system can reveal other contributions to stability, such as nonintuitive minimization of strain elsewhere in the molecule. Indeed, the delicate distance-dependent balance between sterics and London dispersion means that even subtle changes in size and geometry can lead to disparate behavior. Similarly, solvophobic effects also contribute to stabilizing contacts between bulky functional groups, which can be revealed if there is a correlation with the cohesive energy density of the solvent.

## Key References

- AdamC.; YangL.; CockroftS. L.Partitioning Solvophobic and Dispersion Forces in Alkyl and Perfluoroalkyl Cohesion. Angew. Chem., Int. Ed.2015, 54, 1164–116710.1002/anie.20140898225413159.^1^ This work highlights the importance of solvent screening to dissect London dispersion (LD) and solvophobic forces in apolar cohesion.
- WestA. M. L.; Dominelli-WhiteleyN.; SmolyarI. V.; NicholG. S.; CockroftS. L.Experimental Quantification of Halogen···Arene van Der Waals Contacts. Angew. Chem., Int. Ed.2023, 62, e20230968210.1002/anie.202309682PMC1095343837470309.^2^ This work underlines the importance of sterics and electrostatic forces as competitors to LD forces.
- YangL.; BrazierJ. B.; HubbardT. A.; RogersD. M.; CockroftS. L.Can Dispersion Forces Govern Aromatic Stacking in an Organic Solvent?Angew. Chem., Int. Ed.2016, 55, 912–91610.1002/anie.20150805626632979.^3^ In the context of aromatic stacking, drastically varying the size of the rings is essential to discriminate LD forces.
- YangL.; AdamC.; CockroftS. L.Quantifying Solvophobic Effects in Nonpolar Cohesive Interactions. J. Am. Chem. Soc.2015, 137, 10084–1008726159869 10.1021/jacs.5b05736.^4^ This work underlines the importance of cohesive energy density in apolar interactions relative to LD forces in solution.

## 1 Introduction

London forces are the attractive component of van der Waals interactions and can be considered as arising from the spontaneous formation of correlated momentary dipoles in neighboring atoms. The comprehensive examination of London dispersion (LD) forces in solution is challenging due to the inherently weak and competitive nature of these interactions. Notably, LD is additive, which means that greater contact surface areas increase interaction strengths. This phenomenon is exemplified by the melting and boiling points of apolar substances, which tend to increase incrementally with molecular weight. For example, butane and smaller alkanes are gases, pentane to heptadecane are liquids, while octadecane and higher alkanes are solids at room temperature.^5^ However, dispersion rarely occurs as the sole interaction in more complicated molecular recognition processes that occur in solution, and solvation energetics play an intrinsic role in controlling equilibria and kinetics. Similarly, entropy and surface of contact variations arising from different interaction geometries may also make important energetic contributions. This means that significant contextual dependency may arise, which is particularly poignant for weak interactions that do not dominate the overall behavior.

In this Account, we explore the context-dependent significance of LD forces by juxtaposing our experimental results with those of other studies. Specifically, we examine how factors such as the characteristics of the system and the choice of solvents can influence the experimental trends. Our Account commences with an examination of the Wilcox torsion balance, which yielded early insights and at times apparently contradictory interpretations of the significance of LD forces. Subsequently, we summarize the role of LD forces and the context-dependent effects on alkyl–alkyl, halogen−π, alkyl−π, and aromatic stacking interactions.

## 2 The Wilcox Torsion Balance: Context Dependency Revealed by Solvent and Substituent Effects

**Figure 1:**
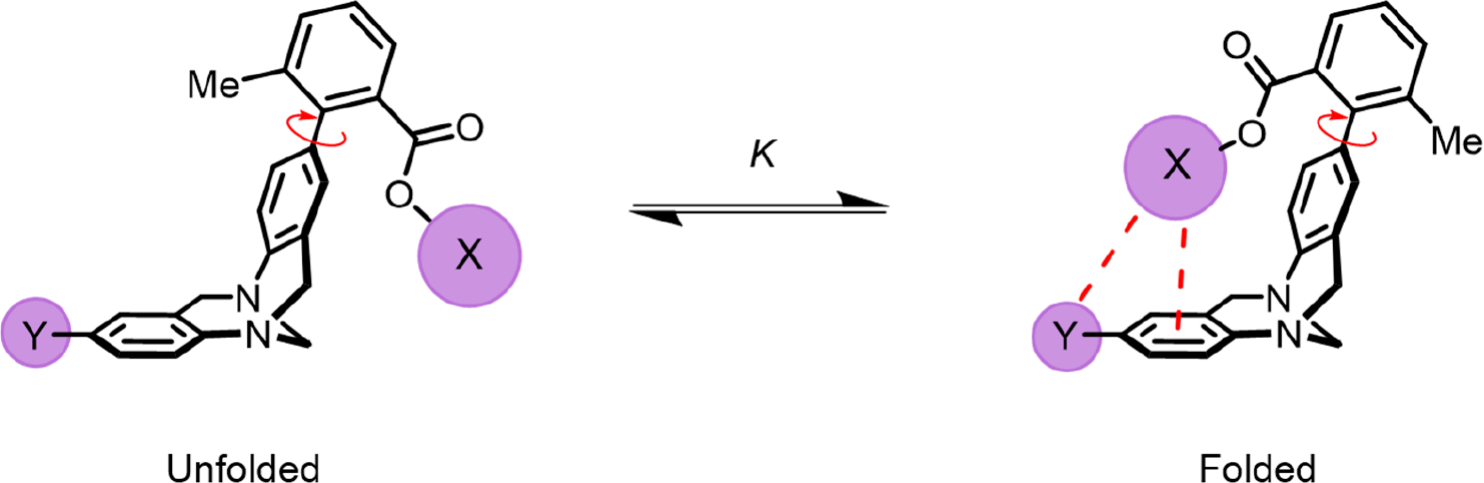
Molecular torsion balance developed by Wilcox and co-workers^6,8^ and later adopted by Diederich^9,10^ and Cockroft^1,4,13,44^ to measure a variety of weak molecular forces.

Wilcox played a pioneering role in the investigation of weak noncovalent interactions. In 1994, he coined the phrase “molecular torsion balance” and proposed that “conformational isomerism can provide a sensitive probe of weak molecular forces”.^6,7^ In his seminal design, restricted rotation around a biaryl bond leads to the observation of distinct and slowly exchanging folded and unfolded conformers in the NMR spectra (Figure 1). In one conformation, an intramolecular aromatic edge-to-face interaction occurs, while in the other this interaction is broken. At the time of this pioneering work, electrostatic interactions were deemed the primary driving force in edge-to-face interactions.^6^ In this work and a follow-up publication,^8^ most of the balances synthesized preferred the folded conformation (0 to −3.4 kJ mol^–1^), with the strongest preference being when X = *t*-Bu. However, when X was a phenyl group, the populations of the folded and unfolded conformations hardly changed as the Y substituent was varied (−1.0 ± 0.2 kJ mol^–1^) in CDCl_3_ solution. Based on these findings, the authors suggested a prominent influence of LD forces in governing the energetics of edge-to-face aryl interactions,^8^ effectively challenging the conventional electrostatic understanding of these interactions.

In contrast, Diederich and co-workers later revealed the expected electrostatic trend when they found that folding energies correlated strongly with the Hammett substituent constants of Y when X = *p*-trifluoromethylphenyl in C_6_D_6_ solution (0 to −4 kJ mol^–1^).^9,10^ While dispersion forces play a role in the folding of these molecular balances, its observation and measurement remain challenging, and it cannot be deemed the dominant driving force in view of the strong electrostatic trend. Cockroft and Hunter later reconciled these contradictory results,^11,12^ and showed that a simple model in which the solvent competes for the edge-to-face interactions could explain the apparently disparate findings. In short, solvation of the Y-substituted phenyl ring by CDCl_3_ in the unfolded conformation is similar in energy to the edge-to-face interaction formed in the folded conformation when X = Ph, so no substituent trend is observed as Y is varied. Meanwhile, the C_6_D_6_ solvent is less able to compete for edge-to-face interactions than the more polar X = *p*-trifluoromethylphenyl group, so the expected electrostatic substituent effect is revealed as Y is varied. The predictions of this simplistic model were later substantiated by independent experimental measurements, thereby confirming that solvation and electrostatic forces exert a governing influence over the observed trends.^10^ Meanwhile, the role of dispersion became less clear, and it appeared that dispersion contributions were similar across all balances examined. At that stage, the lack of data obtained in different solvents meant that it was not possible to distinguish the contributions to folding from LD and solvophobic effects.

**Figure 2:**
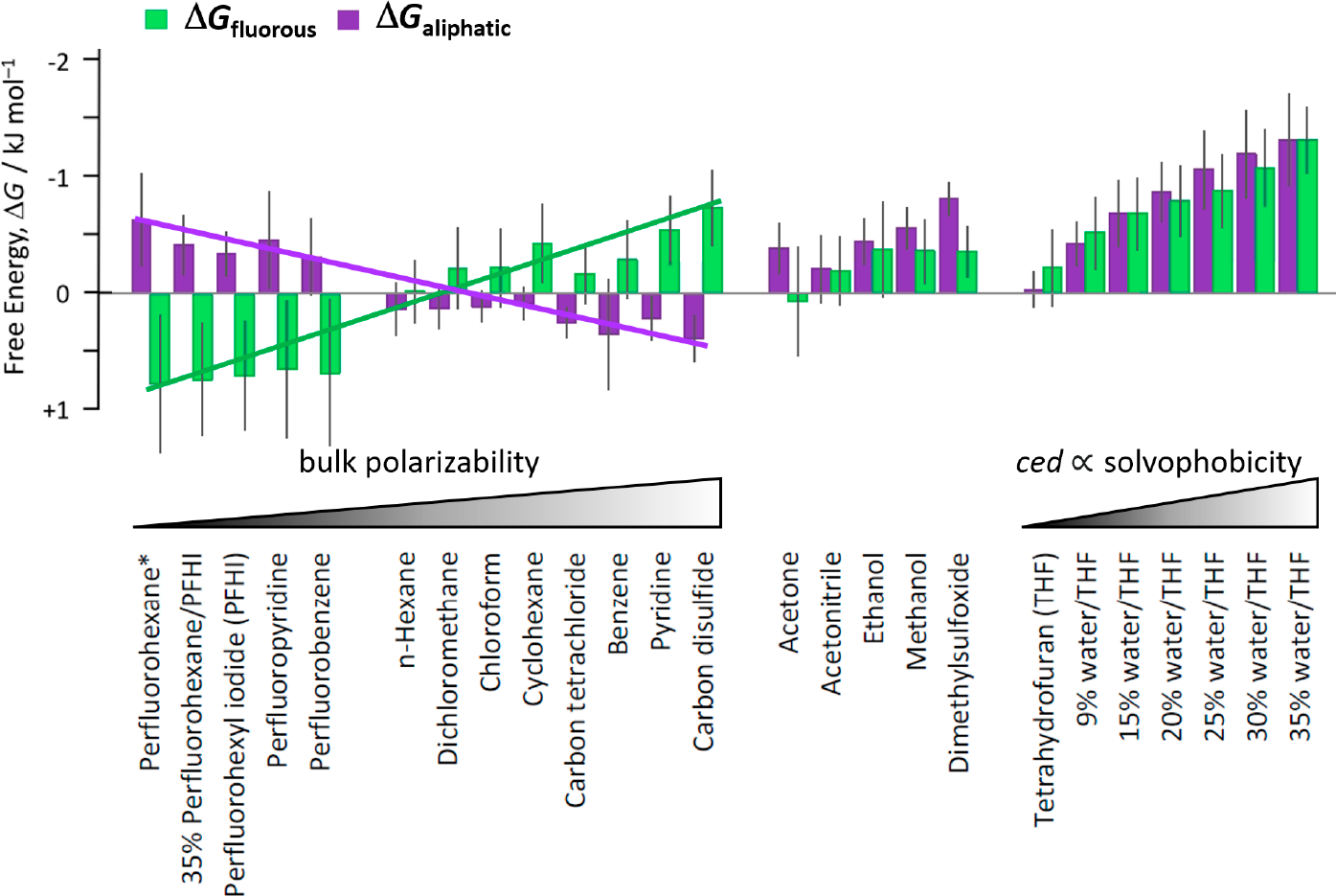
Experimental free energies of alkyl–alkyl (purple) and perfluoralkyl–perfluoroalkyl interactions (green) measured in various solvents using Wilcox torsion balances of the type shown in Figure 1 where X = *n*-heptylphenyl or perfluoro-*n*-heptyl phenyl, Y = *n*-hexylphenyl or perfluoro-*n*-hexylphenyl. Solvent percentages are given as v/v, and *ced* = cohesive energy density. Adapted with permission from ref (1). Copyright 2015 Wiley.

Cockroft and co-workers later deliberately selected Wilcox’s balance to determine the significance of LD relative to solvophobic and electrostatic forces. In light of the prominent role attributed to solvent effects, an extensive screening of solvents was deemed imperative.^1,4,13,44^ Variants of the Wilcox balance were synthesized that featured alkyl and perfluoroalkyl groups (Figure 1, X = *n*-heptylphenyl or perfluoro-*n*-heptylphenyl, Y = *n*-hexylphenyl or perfluoro-*n*-hexylphenyl) to investigate the importance of LD across a spectrum of organic and perfluorinated solvents (Figure 2). Thermodynamic double mutant cycles^14^ were used to dissect the intramolecular alkyl–alkyl and perfluoroalkyl–perfluoroalkyl interaction energies using control compounds in which one or both of the interacting chains were deleted. The experimental interaction energies measured in solution (less than ±1.5 kJ mol^–1^) were an order of magnitude smaller than both the calculated gas-phase energies and the experimental enthalpies of vaporization of the corresponding alkanes and perfluoroalkanes. This indicated that the London dispersion forces between the chains were strongly counterbalanced by competitive dispersion interactions with the surrounding solvent. Experimentally derived bulk solvent polarizability provided a useful measure of the ability of a solvent to engage in competitive dispersion interactions. Interestingly, in apolar solvents alkyl–alkyl contacts shifted from being slightly disfavored to marginally favored as solvent bulk polarizability decreased, but the opposite trend was observed for the perfluoroalkyl–perfluoroalkyl contacts (Figure 2, left). Accordingly, alkyl–alkyl contacts were favored in fluorinated solvents with lower bulk polarizabilities, while perfluoroalkyl–perfluoroalkyl contacts were favored in organic solvents with slightly higher bulk polarizabilities. In more polar solvents, solvophobic effects helped to stabilize both the alkyl and perfluoroalkyl contacts to similar extents, which was most clearly demonstrated by the addition of water to a tetrahydrofuran solution (Figure 2, right). Good correlations were found between the dissected interaction energies and the cohesive energy density of the solvent, which provides an excellent descriptor of solvophobic effects.^4^ These outcomes indicated that both dispersion and solvophobic effects made similar energetic contributions to folding, which was confirmed via a more precise dissection of their contributions using linear regression.

The understanding of solvent competition for dispersion interactions gained from these molecular balance studies also proved useful in predicting the formation of ordered molecular films on surfaces. Evmenenko and Dutta found that ordered polyhedral oligomeric silsesquioxane films could be formed on silica surfaces when the solvent was acetone, THF, or *n*-hexane but not from benzene, toluene, or chloroform.^15^ It was noted that solvents from which ordered films were formed possessed lower Hansen dispersion parameters (δ_D_) and reduced bulk polarizability (or polarizability per unit molecular surface area). This implied that solvents with higher bulk polarizabilities (benzene, toluene, and chloroform) more strongly competed for London dispersion interactions and hindered the assembly of the solute molecules into films. Based on this hypothesis, Cockroft predicted that deposition from the low polarizability solvents hexafluorobenzene and ethyl acetate would also form ordered films, which was indeed confirmed to be the case. These results point toward the general utility of experimental descriptors of solvent polarizability for understanding the behavior of systems where London dispersion plays a guiding role in self-assembly.^1,13,15^

## 3 Context Dependency of Alkyl–Alkyl Interactions

**Figure 3:**
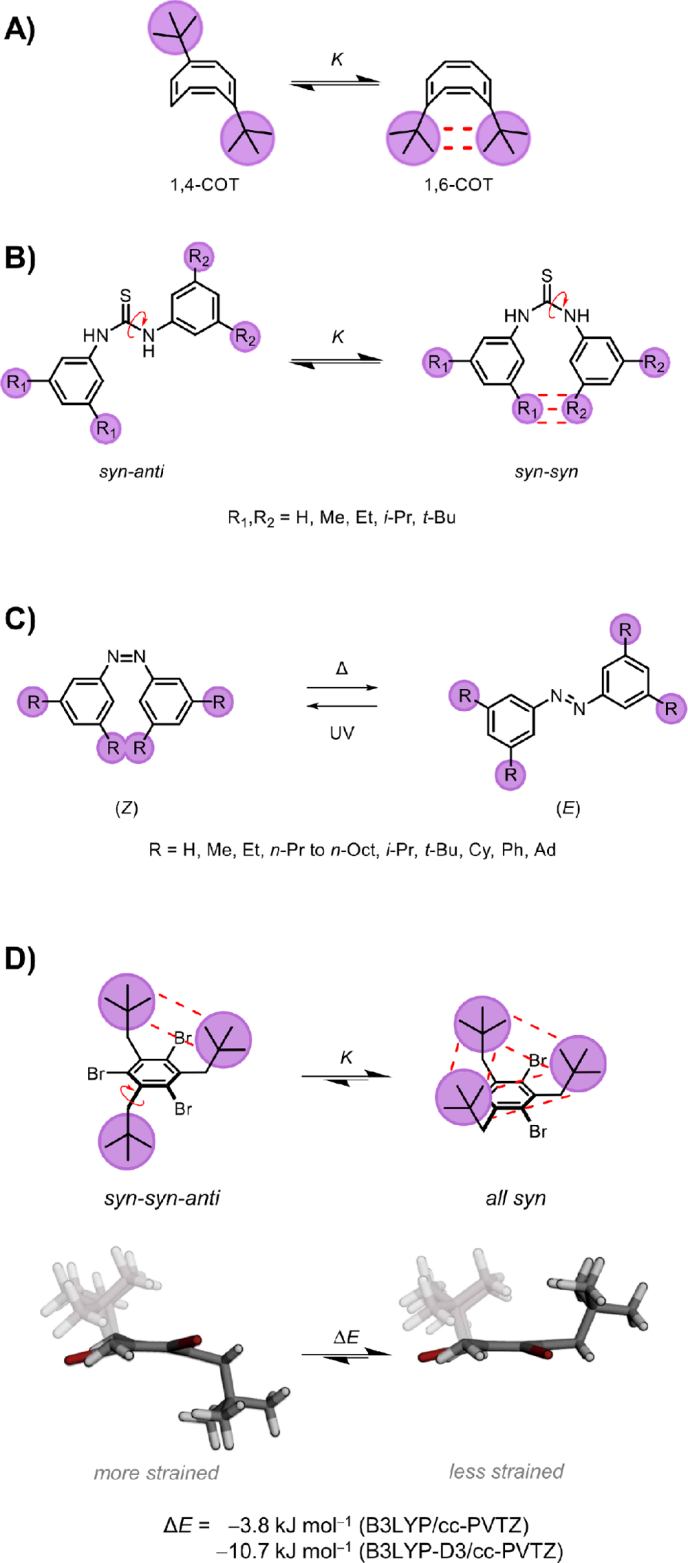
Molecular balances used to examine London dispersion in alkyl–alkyl interactions. (A) Cyclooctatetraene (COT) and (B) diarylthiourea balances used by Schreiner and co-workers.^16,23^ (C) Azobenzene molecular switch used by Wegner and co-workers.^23^ (D) 1,3,5-trineopentylbenzene used by Drakenberg and co-workers.^25^

The Schreiner group have also utilized molecular balances to investigate London dispersion (Figure 3A,B). Balances based on cyclooctatetraene (COT) have been employed to examine LD between alkyl and silyl groups (Figure 3A).^16,17^ One advantage of these systems is the complete lack of polar functionalities, which maximizes the potential for LD contributions to be manifested. Notably, intramolecular van der Waals attraction in the *t*-Bu COT variant had previously been explored computationally by Allinger et al. in 1982, albeit using a now outdated force-field method.^18^ Schreiner’s modern experimental study determined the total free energy, enthalpy, and entropy changes between the two COT isomers in a comprehensive spectrum of solvents. NMR analysis consistently revealed a preference for the apparently more crowded 1,6-isomer in all solvent and temperature combinations examined. Symmetry-Adapted Perturbation Theory (SAPT) calculations^19^ further suggested that this preference could be attributed to the stabilizing influence of London dispersion interactions between the *t*-Bu groups. Intriguingly, the preference for the 1,6-isomer increased as the polarizability of the solvent increased (Catalán–Hopf SP values were used, which correlate strongly with the aforementioned bulk polarizability). This result is in direct contradiction with the results discussed above obtained using Wilcox balances, from which it would be expected that the isomer with the largest solvent accessible area would be preferred in the most polarizable solvents. However, the change in solvent accessible area between the COT isomers is very small (4 Å) compared to the orders of magnitude greater changes associated with folding of the Wilcox balance or bringing longer alkyl or perfluoroalkyl groups into contact. Hence, it should be expected that solvent competition is greatly diminished in the COT system. The difference between these sets of findings highlights the context-dependent impact of solvent accessibility and the effect that this has on solvent competition for dispersion.^1,13,16^

In a follow-up study, Schreiner and co-workers expanded their investigation of COT balances by incorporating silyl groups. Despite the established reputation of these groups as good LD energy donors,^20^ the equilibrium consistently exhibited a shift toward the unfolded 1,4 conformation at varying temperatures, with the trimethyl silane variant standing as the sole exception. The general trend was explained by the voluminous nature of the silyl groups, which were found to strain the core structure of the COT in the 1,6-conformation. SAPT calculations revealed that LD forces stabilized the 1,6-COT conformation, but they proved inadequate to overcome the strain introduced by the voluminous silyl groups. These illustrative cases underscore the delicate balance between steric repulsion and LD forces.^21^ Overall, the COT balances provide excellent examples of the intrinsic contextual sensitivity of LD interactions.

Schreiner and co-workers further investigated LD forces using a molecular balance based on diarylthiourea (Figure 3B).^22^ This design presented three distinct conformations: *anti*–*anti*, *syn*–*anti*/*anti*–*syn*, and *syn*–*syn*, with the latter appearing to be the most sterically hindered. Every balance variant favored the *syn*–*syn* conformation in tetrahydrofuran solution, which was attributed to LD. Moreover, the *syn*–*syn* preference was greatest for those bearing the largest alkyl groups. These results contrast with the behavior of silyl substituted COT where steric hindrance was not compensated by LD forces.^20^ However, it is imperative to underscore that this study was limited to THF, and solvents with different properties might have exerted a significant influence on the outcomes. Additionally, the exploration primarily focused on relatively small alkyl groups.

Wegner and co-workers directed their focus toward studying the influence of LD forces on the *Z* to *E* isomerization rates of azobenzene switches functionalized with short linear, cyclic, and branched alkyl groups (Figure 3C).^23,24^ This investigation encompassed measurement of the isomerization rates in dimethyl sulfoxide (DMSO) and *n*-octane. As the bulkiness of substituents increased, the rate of isomerization away from the *Z* isomer exhibited a corresponding decrease. The most pronounced effect was observed with the adamantyl-substituted azobenzene, which was consistent with LD forces being most potent for the bulkier substituents that were able to form better intramolecular contacts. However, different results were obtained in a subsequent study that explored the influence of long linear alkyl chains (methyl to *n*-octyl) in solvents spanning *n*-heptane to *n*-dodecane, iso-octane, and cyclooctane.^24^ The solvent did not significantly impact intramolecular LD interactions, and similar trends were observed across different solvent environments. Although the inherent flexibility of both the long alkyl substituents and *n*-alkane solvents introduced variability in the observed half-lives of the *Z* isomers, only marginal variation was observed for the *n*-propyl to *n*-octyl balances. Most strikingly, the results were found to correlate with the surface tension of the respective solvents. Cohesive tension is a descriptor of solvent cohesive effects,^4^ but in contrast, Cockroft and co-workers found the energetics of polystyrene folding, edge-to-face and face-to-face aromatic stacking, alkyl–alkyl, and perfluoroalkyl–perfluoroalkyl interactions all correlated better with the cohesive energy density (CED) of the solvent rather than surface tension.^1,4^

On first inspection, the findings of Schreiner and Wenger seem commensurate with those of Drakenberg who determined that 1,3,5-trineopentylbenzene exhibits a pronounced preference for what appears to be the most sterically hindered all-*syn* conformation (Figure 3D).^25^ This suggests that the LD forces stabilize the all-*syn* conformation. However, counterintuitively, examination of the computationally minimized structures (Figure 3D, bottom) reveals that the all-*syn* conformer is less distorted away from planarity compared to the *syn*–*syn*–*anti* conformer. Accordingly, gas-phase calculations indicate that the “most sterically hindered” all-*syn* conformer is, in fact, least strained and most stable, irrespective of whether dispersion is included (B3LYP-D3/cc-PVTZ vs. B3LYP/cc-PVTZ). Nonetheless, the difference in the energy between these two sets of calculations indicates that strain release accounts for one-third of the stabilization of the all-*syn* conformer with two-thirds from dispersion. Once again this underlines the context dependency of LD forces, while also raising the importance of thoroughly understanding all physicochemical aspects contributing to the relative stability of isomers.

## 4 Context Dependency of Interactions with Aromatic Faces (Alkyl–Arene, Halogen–Arene)

**Figure 4:**
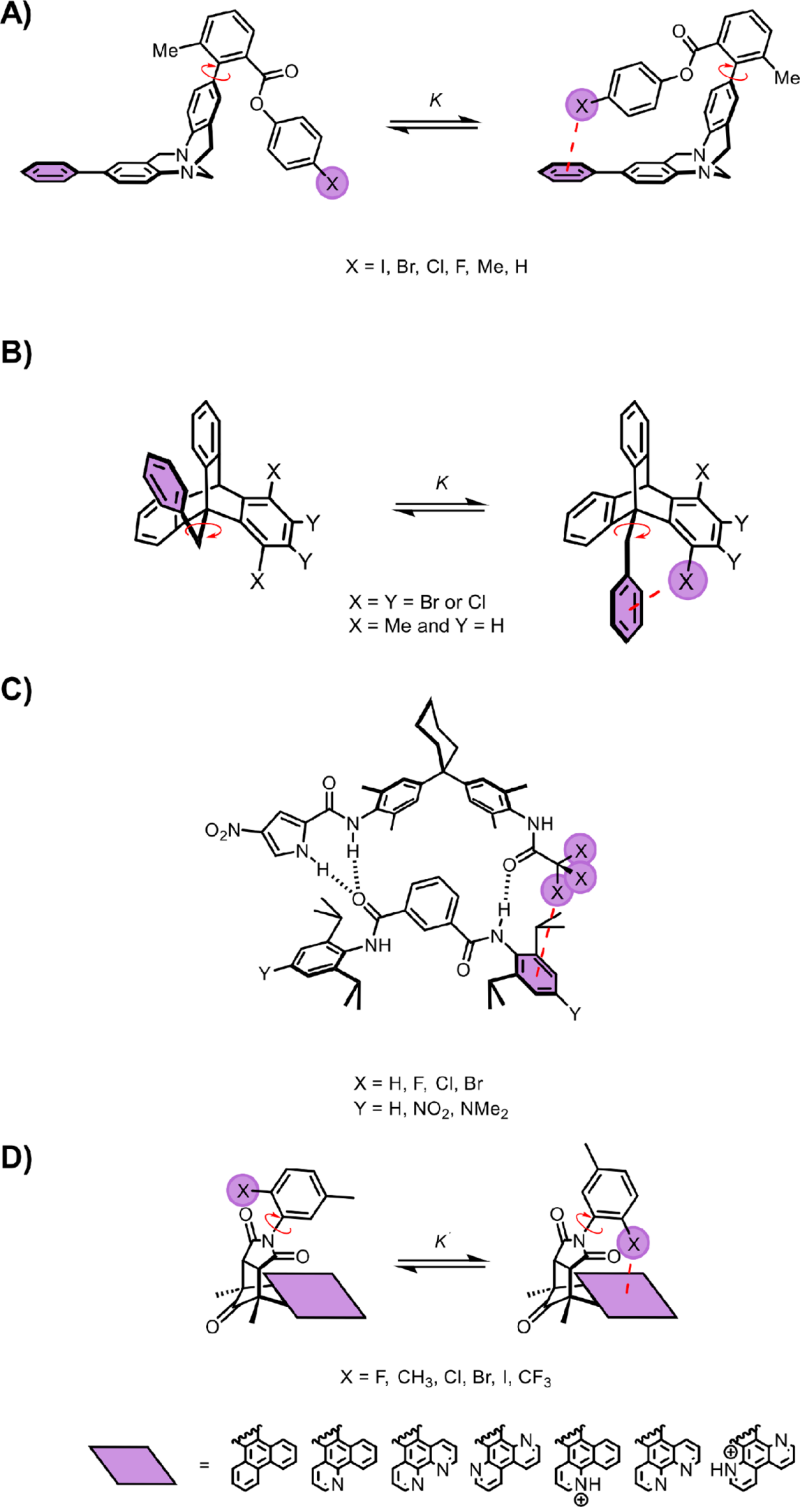
Synthetic supramolecular systems for investigating halogen and alkyl interactions with the faces of aromatic rings. (A) Wilcox torsion balance used by Cockroft and co-workers.^2^ (B) Trypticene torsion balance used by Oki and co-workers.^26^ (C) H-bonded “zipper” complex used by Hunter and co-workers.^28^ (D) Shimizu *N*-arylimide molecular torsion balance.^29^

Wilcox’s balance was originally designed to examine edge-to-face aromatic interactions, but the scaffold has also permitted the study of other interactions. For example, Cockroft and co-workers used the modified balance design shown in Figure 4A to study halogen–arene interactions.^2^ This molecular balance was chosen to place halogen atoms in contact with aromatic rings and to compare such halogen–arene interactions with CH_3_–arene interactions. The choice of the Wilcox balance design stemmed from its intrinsic flexibility, which enabled the varied steric demands of the halogens to be accommodated. Thermodynamic double mutant cycles enabled the dissection of the halogen–arene interaction energies in 17 solvents and solvent mixtures. Surprisingly, the halogen–arene interactions were slightly disfavored across all 17 solvents, while the methyl–arene interactions were slightly favored. Computational energetic dissection using SAPT revealed a less favorable electrostatic component in the halogen–arene interactions compared to methyl–arene interactions.^19^ Experimentally, the halogen–arene interaction became more disfavored as the size of the halogen increased, which contrasted with the SAPT prediction that the interaction of the phenyl ring with the larger, more polarizable iodine substituent would be the most favored. The implication was that solvent competition for dispersion reduced this potentially favorable contribution to folding. Nonetheless, solvents with either lower polarizabilities or high solvophobicity tended to make the halogen–arene interaction slightly less disfavored, though never as favorable as the CH_3_–arene interaction. Hence, it was discerned that the intramolecular LD forces in this system, even those between polarizable groups such as an iodine substituent and a phenyl ring, lacked the potency to stabilize the folded conformation in solution.

Halogen–arene and CH_3_–arene interactions in side-on geometries have also been investigated in seminal works by Oki^26^ and Schneider.^27^ Similar to the findings determined using the Wilcox balance shown in Figure 4A,^2^ Oki’s early studies using triptycene molecular balances (Figure 4B found Cl–arene and Br–arene interactions to be repulsive but, in contrast, less repulsive than CH_3_–arene interactions. Meanwhile, Schneider used porphyrin complexes with a series of smaller aromatic guests^27^ and found that the complexes became more stable as the polarizability of the substituents increased (i.e., increased in the order F < Me < Cl < Br < I). The correlation between complexation energies and polarizability of a wide range of substituents pointed to the importance of dispersion in the association of the complex. However, the interaction geometry of the complexes was unknown. Moreover, the association constants decreased by more than 3 orders of magnitude when titrations were performed in a range of ethanol/water mixtures, and the data was very highly correlated (*R*^2^ = 0.995) with the solvophobicity parameter (*S*_p_) of the solvent. This latter result once again points to the challenge of dissecting solvophobic and dispersion interactions, which both increase as the size (and hence atomic polarizability) of the substituents increase.^1,2,4,13^

These investigations examined side-on halogen–arene interactions. Other studies have examined halogen–arene interactions in end-on geometries that could potentially facilitate halogen bonding by aligning the electron-deficient σ-hole of a halogen atom with an electron-rich aromatic face. For example, Hunter investigated halogen–arene interactions using an H-bonded “zipper” complex (Figure 4C),^28^ while Shimizu’s molecular balance has assessed such interactions in an intramolecular context (Figure 4D).^29−31^ Irrespective of whether the halogen–arene contact was in a side-on or end-on geometry, the interaction trends tended to be dominated by steric effects. An important exception was identified by Shimizu et al. for variants facilitating contacts between the organic fluorine atoms and electron-deficient and cationic aromatic surfaces. These attractive fluorine–arene interactions were indicated to be dominated by electrostatics rather than dispersion. The contributions from solvophobic effects were likely to be minimal since the experiments were performed in a CD_2_Cl_2_ solution. Most pertinently, Shimizu and co-workers have measured a wide-range of functional group–arene interactions in the same class of molecular balance, which facilitates the comparison of their relative stabilities.^32^ Consistent with the findings determined in the Wilcox balance,^2^ halogen–arene interactions with phenyl rings were found to be repulsive, while the CH_3_–arene interactions were weakly favorable.^33^

## 5 Context Dependency of Aromatic Stacking

**Figure 5:**
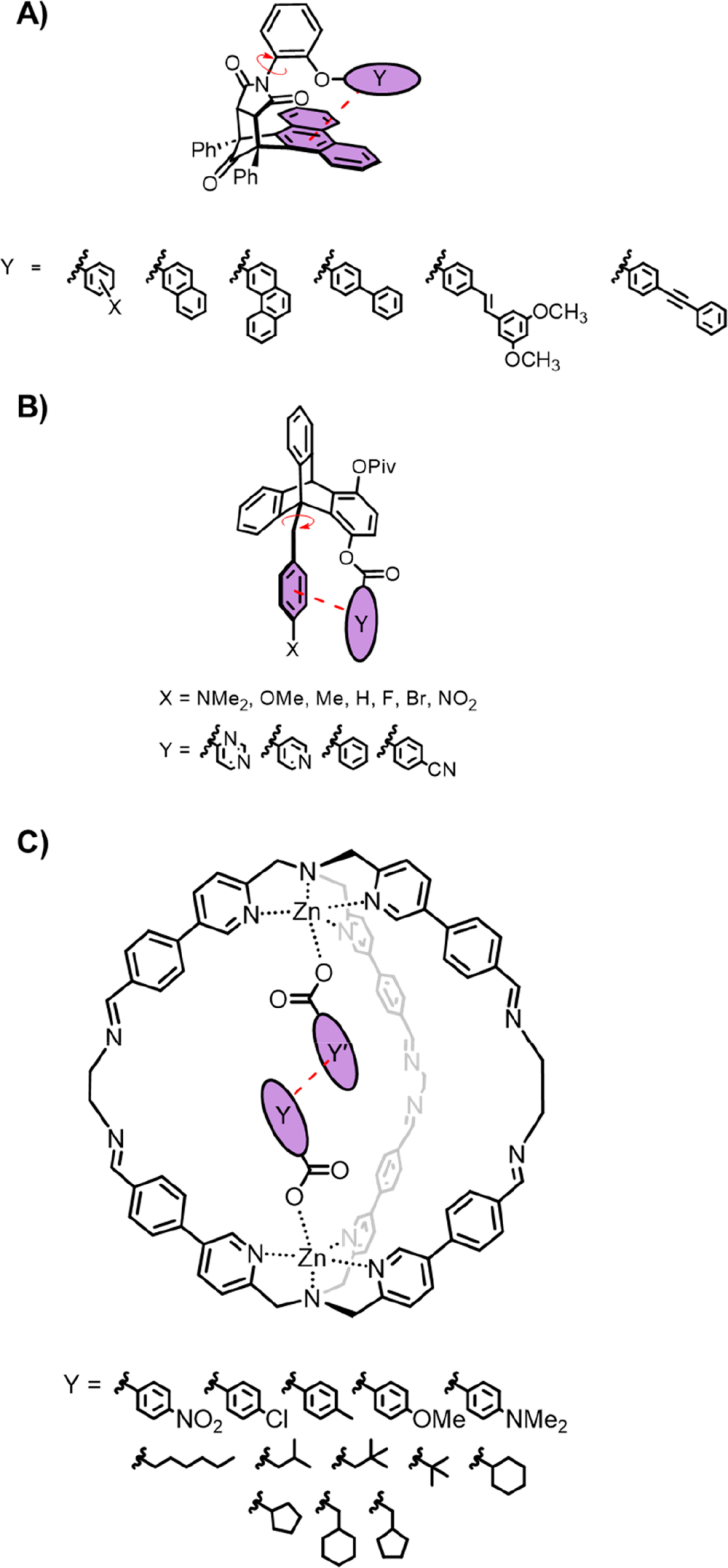
Molecular balances and complexes used to investigate aromatic stacking interactions. (A) *N*-arylimide molecular torsion balance used by Shimizu and co-workers.^34,35^ (B) Trypticene torsion balance used by Gung and co-workers.^37,38^ (C) Ternary complex used by Zonta and co-workers^40^ to measure aromatic stacking interactions relative to alkyl–arene interactions.

Shimizu and co-workers have also used molecular balances to examine aromatic stacking interactions (Figure 5A).^34,35^ Balances containing differently substituted phenyl ethers revealed the anticipated electrostatic substituent effects. Stacking was more favorable than a CH_3_–arene interaction when the X substituent was electron-withdrawing, but less favorable than a CH_3_–arene interaction when the X substituent was electron-donating.^35^ A diverse range of aromatic moieties of varying sizes and hence polarizabilities were examined to probe the role of LD.^33^ However, the size and polarizability of the aromatic surfaces exhibited minimal impacts on the resultant folding energies in solution. The authors attributed this minimal influence to the cancellation of dispersion by solvent competition. While such cancellation undoubtedly occurs,^13^ another potential cause might be that the area of stacked contact varied little between the different balances. Indeed, molecular polarizability is approximately atomically additive,^36^ so while the total polarizability of the larger aromatics was increased, the local polarizability of the aromatic faces will be similar in all of the stacking contacts.

**Figure 6:**
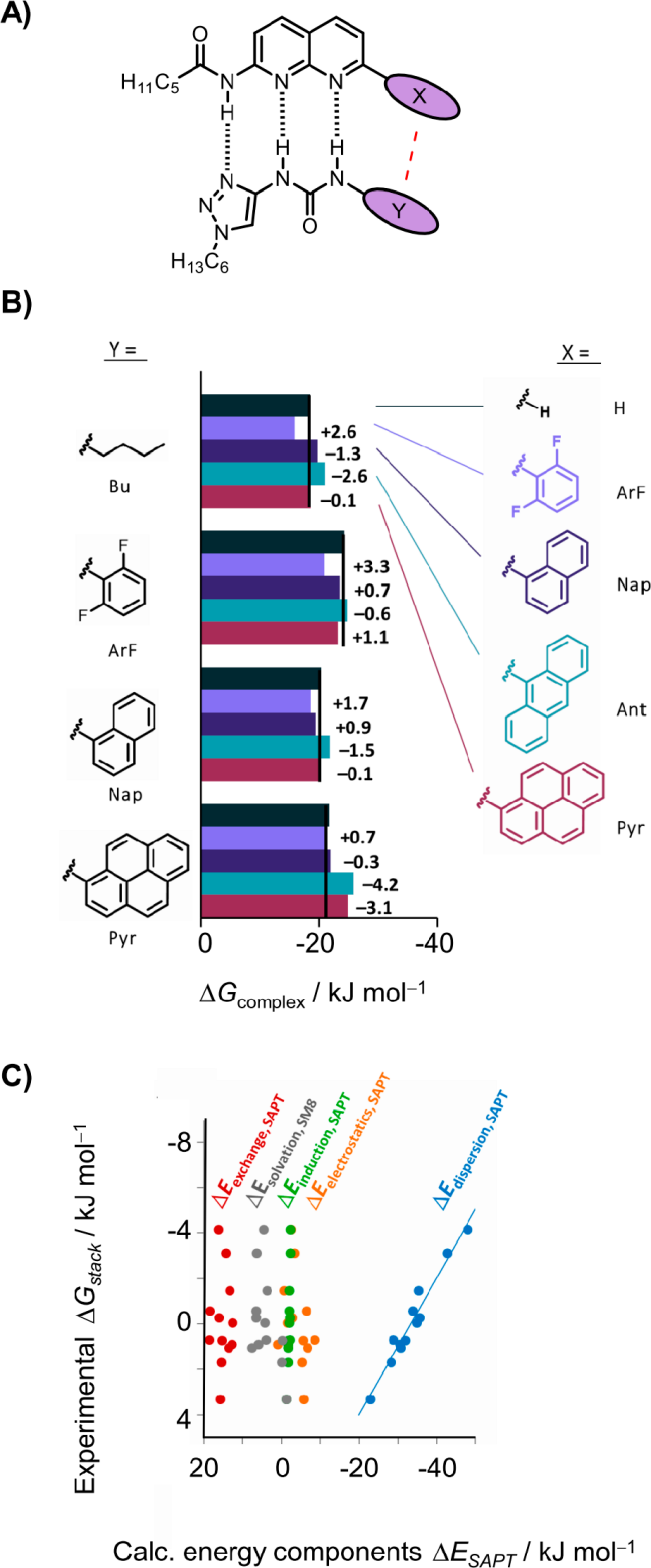
(A) H-bonded complex used by Cockroft and co-workers to examine aromatic stacking interactions. (B) Experimental Δ*G* complexation energies measured in 5% MeCN/CDCl_3_. (C) SAPT calculated energy components plotted against experimental dissected Δ*G* stacking interactions (using the X = H compound as a reference). Panels B and C were adapted with permission from ref (3). Copyright 2016 Wiley.

Gung and co-workers similarly used triptycene molecular balances to investigate aromatic stacking (Figure 5B).^37^ The initial results were consistent with electrostatic interactions primarily governing these stacking interactions.^7,37^ However, pyridine and pyrimidine substituted triptycenes were found to host stronger stacking interactions with phenyl rings than the corresponding non-heterocyclic phenyl–phenyl interactions (Figure 5B).^38^ The authors suggested LD, local dipole–dipole, and donor–acceptor interactions as possible explanations for the observed results. However, the areas of aromatic stacking contacts again varied little between the different balances. Hence, Cockroft and co-workers set out to investigate the role of LD on stacking in solution using complexes in which there was larger variation in the stacking contact area (Figure 6A).^37^ Many of the stacking interactions made small or slightly repulsive contributions to complexation, but these interactions became stabilizing for larger contacts. Moreover, the stacking interaction energies were found to correlate with the change in the solvent accessible area upon complexation (i.e., the size of the stacked aromatic contact). The most favorable interaction was found for the anthracene–pyrene stack (−4.2 kJ mol^–1^, Figure 6B). Most strikingly, the experimental stacking interaction energies only correlated with the dispersion component of the calculated SAPT energy components and not the electrostatic, induction, or exchange terms (Figure 6C). As it was highlighted earlier, investigating LD requires a large solvent span in order to fully determine the nature of the interactions.^4^ However, only one solvent mixture was used in this study due to solubility limitations and the requirement for measuring binding over a millimolar concentration range by ^1^H NMR spectroscopy. Nonetheless, it was still possible to show by comparison with Iverson’s prior investigation of solvent effects on aromatic stacking^39^ that solvophobic effects made only a minor contribution to the measured stacking interaction energies in the CDCl_3_/CD_3_CN solvent mixture employed. Hence, it was possible to determine that LD dispersion could indeed govern aromatic stacking in an organic solvent, even in the presence of significant solvent competition. Mirroring the discussion in the previous section, the same class of complex was also used to measure alkyl–arene interactions, which were found to be more favorable than stacking for comparable contact areas.^3^ Again, SAPT calculations indicated that this difference originated from additional electrostatic interactions in the case of the alkyl–arene interactions rather than a difference in the LD interactions.

It is interesting to compare the above examinations of aromatic stacking and alkyl–arene interactions with the recent work of Zonta (Figure 5C).^40^ Experiments were performed in which variously substituted benzoic acids competed against aliphatic carboxylic acids for binding at the two tris(pyridylmethyl)amine sites. ^1^H NMR spectroscopy could be used to determine the population of states for different combinations of acids since the bound states were in slow exchange. Thermodynamic double mutant cycle analysis was then performed to determine the relative stabilities of the various aromatic stacking and alkyl–arene interactions within the core of the complex. Consistent with the studies discussed earlier, the aromatic stacking interactions were most attractive when the substituents on both rings were electron-withdrawing (−4.5 kJ mol^–1^), and most repulsive when both substituents were electron-donating (+2.5 kJ mol^–1^). The authors then extrapolated electrostatic contributions using σ_m_ Hammett parameters to reveal the underlying nonpolar contributions to aromatic stacking relative to several different alkyl–arene contacts. Nonpolar contributions to stacking, which can likely be assumed as corresponding to LD forces, were determined to range from −6 to −3 kJ mol^–1^, while surprisingly, the polar contribution was found to be unfavorable in all cases. The aromatic stacking interaction outcompeted the alkyl–arene interactions in all cases, which contrasts with studies outlined earlier in this section. Interestingly, cyclic alkyl–arene interactions competed for aromatic stacking slightly less strongly (1 kJ mol^–1^) than did acyclic alkyl–arene interactions. The contrasting results on the relative strengths of aromatic stacking and alkyl–arene interactions again highlights the context-dependency of interactions in different systems in which the geometries of the interactions differ. Nonetheless, the results in Zonta’s study further underscore the occurrence of very substantial but imperfect cancellation of LD forces in molecular recognition processes occurring in solution.

## 6 Conclusions

The ubiquitous nature of LD forces demands their consideration in all investigations, whether conducted computationally, in solution, or in the solid state. However, the outcomes of investigations of LD are profoundly influenced by their context: the nature and geometry of the interacting species, whether intra- or intermolecular, and the solvent or lack of it. The significance of LD interactions in the molecular recognition process is strongly attenuated by competition with the surrounding solvent. The bulk polarizability of a solvent can be used to assess the maximal extent of such cancellation in scenarios where there a recognition event involves large changes in the solvent accessible area. However, solvent accessibility varies from system to system, which results in variable extents of solvent cancellation in different contexts.^1^ For example, substantial variation in the size of aromatic contacts is essential to invoke significant energetic changes arising from LD.^34,3^ Since both LD and solvophobic contributions scale with the sizes of the interacting species, this makes determining their relative significance challenging,^1,40^ and large-scale solvent screening is often required.^9,4,39^ However, few systems are soluble in a wide range of solvents, and if they are soluble, it indicates that the solvent is competing with the very interactions that one is seeking to investigate. Indeed, it is difficult for intermolecular complexation in solution to be driven by weak residual LD interactions, which means that it is usually a requirement that LD contributions are measured as a perturbation of other stronger interactions. Intramolecular systems such as molecular balances^7^ can overcome at least the bimolecular association entropy cost and aid in the examination of weak interactions such as dispersion. However, the use of intramolecular systems brings other context-dependent caveats, such as steric strain.^20,25^ Meanwhile, the geometries of specific functional group interactions in intramolecular contexts may differ from those of intermolecular complexes, which may be closer to the isolated equilibrium separation and geometry. In addition, electron delocalization via induction/polarization makes larger contributions to molecular interactions at short separations, which may further cloud the view of LD.^2,4,41−43^ More generally, electrostatic interactions frequently dominate over LD since their energies scale with a 1/*r*^2^ distance dependence, while dispersion has ∼1/*r*^6^ dependence. The contrasting findings of the experimental studies of alkyl–alkyl, alkyl–arene, halogen–arene, and aromatic stacking interactions presented in this Account provide very good examples where the role of LD has proven to be challenging to isolate, due in part to contextual dependency.

Due to the experimental challenge of discerning the significance of LD interactions against multiple background contributions, it is increasingly common for studies of LD to compare experimental results with computationally determined geometries and energies. Such comparisons reveal that LD forces are attenuated by approximately an order of magnitude due to solvent competition compared to the gas phase, but such cancellation is not universally transferable.^1,3,44^ For example, dispersion-corrected calculations predict the folding energies of Wilcox balances hosting alkyl···alkyl and perfluoroalkyl···perfluoroalkyl interactions to lie between −17 to −40 kJ mol^–1^ in the gas phase, but the experimental folding energies in solution are no greater than −4 kJ mol^–1^.^4^ Thermodynamic double mutant cycle analysis of the same energies predicts that dispersion between the chains contributes −13 to −20 kJ mol^–1^ to folding in the gas phase. However, analysis of the experimental energies reveals that the alkyl–alkyl and fluoroalkyl–fluoroalkyl interactions make unfavorable contributions of up to +1.5 kJ mol^–1^, i.e., solvation of the chains is favored over the interactions between the chains. Such disparate findings contrast with those of Schreiner and co-workers who found that *tert*-butyl···*tert*-butyl contacts contribute up to −1.9 kJ mol^–1^ to the folding of cyclooctatetraene molecules in solution, which was similar to the value of up to −3.3 kJ mol^–1^ calculated for the gas phase.^16^ The implication is that the solvent accessibility and hence extent of solvent competition for dispersion differ greatly between the Wilcox balance and the much smaller cyclooctatetraene frameworks. Indeed, large changes in the apolar surface area upon binding or folding are required for dispersion-driven energetic influences to manifest.

True energetic comparisons between experiment and theory cannot be made, because computational solvent models are underdeveloped and inaccurate. Nonetheless, even when constrained to the gas phase, computational energy partitioning methods, such as SAPT^19^ and energy decomposition analysis,^45,46^ provide valuable insights into the relative energetic contributions of electrostatics, induction, exchange repulsion, and dispersion, which are otherwise difficult to assess experimentally. Indeed, experimental measurements of molecular recognition events in solution provide an avenue to develop computational methods with improved solvent models. Data obtained from molecular balances may be particularly useful for benchmarking computational solvent models since interaction geometries are well-defined, and wide-range screening of solvent effects is experimentally viable. It will only be through the powerful pairing of experiment and theory that we will be able to solve the challenges of solvent and context dependency in molecular recognition phenomena.
